# Arachidin-1, a Prenylated Stilbenoid from Peanut, Induces Apoptosis in Triple-Negative Breast Cancer Cells

**DOI:** 10.3390/ijms23031139

**Published:** 2022-01-20

**Authors:** Sepideh Mohammadhosseinpour, Linh-Chi Ho, Lingling Fang, Jianfeng Xu, Fabricio Medina-Bolivar

**Affiliations:** 1 Molecular Biosciences Graduate Program, College of Sciences and Mathematics, Arkansas State University, Jonesboro, AR 72467, USA; sepideh.mohammad@smail.astate.edu; 2 Arkansas Biosciences Institute, Arkansas State University, Jonesboro, AR 72467, USA; lho@uams.edu (L.-C.H.); lfang@astate.edu (L.F.); jxu@astate.edu (J.X.); 3 College of Agriculture, Arkansas State University, Jonesboro, AR 72467, USA; 4 Department of Biological Sciences, Arkansas State University, Jonesboro, AR 72467, USA

**Keywords:** triple-negative breast cancer, peanut, hairy roots, stilbenoids, prenylation, resveratrol, arachidin-1, arachidin-3, cell viability, apoptosis, flow cytometry

## Abstract

Triple-negative breast cancer (TNBC) is unresponsive to typical hormonal treatments, causing it to be one of the deadliest forms of breast cancer. Investigating alternative therapies to increase survival rates for this disease is essential. The goal of this study was to assess cytotoxicity and apoptosis mechanisms of prenylated stilbenoids in TNBC cells. The prenylated stilbenoids arachidin-1 (A-1) and arachidin-3 (A-3) are analogs of resveratrol (RES) produced in peanut upon biotic stress. The anticancer activity of A-1 and A-3 isolated from peanut hairy root cultures was determined in TNBC cell lines MDA-MB-231 and MDA-MB-436. After 24 h of treatment, A-1 exhibited higher cytotoxicity than A-3 and RES with approximately 11-fold and six-fold lower IC_50_, respectively, in MDA-MB-231 cells, and nine-fold and eight-fold lower IC_50_, respectively, in MDA-MB-436 cells. A-1 did not show significant cytotoxicity in the non-cancerous cell line MCF-10A. While A-1 blocked cell division in G2-M phases in the TNBC cells, it did not affect cell division in MCF-10A cells. Furthermore, A-1 induced caspase-dependent apoptosis through the intrinsic pathway by activating caspase-9 and PARP cleavage, and inhibiting survivin. In conclusion, A-1 merits further research as a potential lead molecule for the treatment of TNBC.

## 1. Introduction

Breast cancer is a major cause of cancer-related death among women in the United States. It is divided into four main subtypes: luminal A; luminal B; triple-negative/basal-like; and human epidermal growth factor receptor 2 (HER2)-enriched [1]. Among these, 15% of cases are triple-negative breast cancer (TNBC), the most aggressive and fatal type of cancer due to the lack of all three receptors (estrogen receptors/progesterone receptors/HER2) [2]. The most common form of treatment for TNBC is chemotherapy. However, TNBC cells are prone to developing multi-drug resistance to chemotherapy drugs and around 90% of these drugs are highly toxic to non-cancerous cells, which can cause severe side effects, including congestive heart failure, hair loss, nausea, and joint and muscle pain [1,2,3,4]. Therefore, there is an ongoing need to find a new treatment for TNBC.

**Figure 1:**
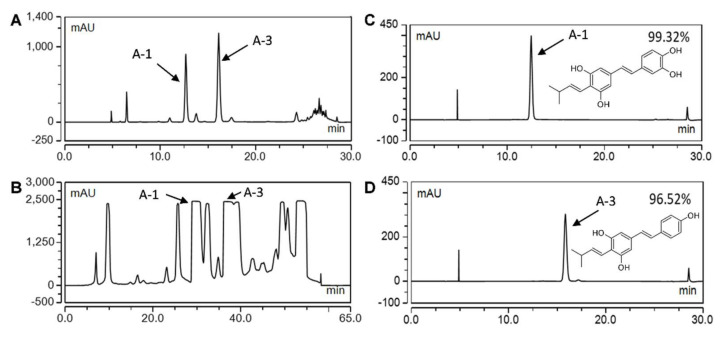
HPLC chromatograms (UV 340 nm) of extracts and purified prenylated stilbenoids from the medium of elicited peanut hairy root cultures. (**A**) Analytical HPLC chromatogram of extract from the culture medium showing arachidin-1 (A-1) and arachidin-3 (A-3). (**B**) Semi-preparative HPLC chromatogram of extract from the culture medium. (**C**) Purified A-1 and chemical structure. (**D**) Purified A-3 and chemical structure.

Natural products, such as stilbenoids, play an important role in cancer research [5]. Stilbenoids are specialized metabolites found in various plants, such as grapevine (*Vitis vinifera*), pine (*Pinus*), blueberry (*Vaccinium* spp.), and peanut (*Arachis hypogaea*) [6]. When exposed to biotic stresses, these plants accumulate stilbenoids as phytoalexins, which serve as a defense mechanism [7]. In peanut plants, most of the stilbenoids are prenylated, containing a prenyl group or prenylated derivative at the C-4 position of the stilbene backbone, such as in arachidin-1 (A-1) and arachidin-3 (A-3; Figure 1). To produce these prenylated stilbenoids, peanut hairy root culture systems have been established as an elicitor-controlled bioproduction platform for these potential bioactive compounds [8,9,10].

The non-prenylated stilbenoid resveratrol (RES) has been the most studied stilbenoid and several studies have demonstrated both its anticancer activity and multiple health-promoting properties [6]. Indeed, several in vitro and in vivo studies show that RES exhibits anti-inflammatory, antioxidant, cardioprotective, antiviral, and antiaging properties. RES showed anticancer activity in breast cancer cells by arresting the cell cycle, damaging cancer cell DNA, and blocking angiogenesis and cell proliferation by modulation of transcription factors [11,12]. RES also induced programmed cell death through apoptosis in breast cancer cells [13,14].

Apoptosis is essential for targeting therapeutic agents in cancer cells [15]. It occurs through two main pathways: extrinsic (death receptor-mediated) and intrinsic (mitochondria-mediated). The extrinsic and intrinsic pathways activate caspase-8 and 9, respectively. Caspase-9 is activated by apoptotic protease activating factor-1 (Apaf-1) [16]. Apaf-1 is a molecule that binds to cytochrome c and dATP to form an apoptosome complex, which cleaves procaspase-9 and releases an activated form of caspase-9 [16]. Caspase-8 and 9 are initiators for apoptotic cascades that activate downstream caspases-3, 6, and 7 [16]. PARP is cleaved through caspase-3. Therefore, activation of PARP reduces cell viability and induces apoptosis [17]. Survivin is an apoptosis inhibitor and increases cancer cell resistance to therapy. RES downregulated the survivin protein level in MDA-MB-231 by activating sirtuin 1, which caused activation of caspase-3 and 7. RES induced apoptosis in breast cancer tumor cells by activating apoptosis regulator BAX (Bcl-2-associated X protein) and caspase-3 [18]. RES, alone and in combination with other drugs or natural products, decreased drug resistance and increased the expression of PARP, caspase-3, 8, and 9 in MDA-MB-231 and MDA-MB-436 TNBC cell lines [19,20,21,22,23,24]. Piceatannol, a hydroxylated RES analog, showed PARP activation in human leukemia HL-60 cells [25]. Although RES showed activation of caspase-8, it mainly showed apoptosis induction through the intrinsic pathway by activating caspase-9 [26,27,28,29,30]. Additionally, RES has been tested on MDA-MB-231 and MDA-MB-436 cells, and these cell lines displayed inhibited proliferation due to a reduction in cell division, effectively altering cell cycle phases [31].

Due to the low bioavailability of RES, many studies focus on identifying alternative resveratrol analogs with better pharmacokinetic properties. Prenylated stilbenoids may exhibit enhanced bioavailability when compared to RES due to the prenyl group. Brents et al. (2012) demonstrated the potential high bioavailability of the prenylated stilbenoids A-1 and A-3 via their slower metabolism in vitro [32]. A-1 and A-3, the most abundant prenylated stilbenoids found in elicited peanut hairy root cultures [9], produced lower glucuronidated products when compared to their non-prenylated analogs piceatannol and RES, respectively [32]. Furthermore, the prenyl group increases the hydrophobicity of the stilbenoid and thus may improve its interaction with the cell membrane.

The extra hydroxyl group in A-1, when compared to RES, also provides antioxidant and antiproliferation effects [33]. There are few studies about the anticancer activity of A-1 and A-3. Still, in one such study using human acute promyelocytic leukemia cell line HL-60, A-1 purified from germinated peanut seeds showed increased cell depolarization, which may facilitate uptake by the mitochondrial membrane in either a caspase-dependent or independent manner, causing programmed cell death [34]. In this cell line, A-1 showed higher cytotoxicity than A-3 and apoptosis induction through the intrinsic apoptotic pathway, possibly due to its prenyl group [34]. However, the anticancer activity of A-1 and A-3 remains unknown in other cancer cell lines mainly because these compounds are not commercially available. By using the peanut hairy root cultures as a high-level and sustainable bioproduction system for A-1 and A-3, these compounds can be produced and purified at amounts sufficient for different assays in vitro and in vivo.

The aim of this study was to understand the effects of the natural prenylated stilbenoids A-1 and A-3 on TNBC proliferation and apoptosis induction, and to investigate their impact on the expression of apoptosis-related marker proteins. To this end, the specific objectives were to purify A-1 and A-3 from elicited hairy root cultures and assess their anticancer effects on TNBC cell lines by examining cytotoxicity, intrinsic and extrinsic-mediated apoptosis, and cell cycle arresting.

## 2. Results

### 2.1. Bioproduction and Purification of Prenylated Stilbenoids from Peanut Hairy Root Cultures

Previously established hairy root cultures of peanut cv. Hull line 3 were used as a bioproduction system for prenylated stilbenoids [35,36,37]. Upon co-treatment of the hairy root cultures with methyl jasmonate (MeJA), cyclodextrin (CD), hydrogen peroxide, and magnesium chloride, the hairy roots secrete stilbenoids into the culture medium [36]. Analytical HPLC analyses show high levels of A-1 (UV absorption maxima of 341 nm) and A-3 (UV absorption maxima of 336 nm) in the elicited medium (Figure 1A). To purify A-1 and A-3, ethyl acetate extracts pooled from several hairy root cultures were concentrated and separated by semi-preparative HPLC (Figure 1B). The recovery rates of A-1 and A-3 from the culture medium were 57.08% and 50.15%, respectively. Purified A-1 and A-3 fractions with 99.32% and 96.52% of purities, respectively, were used for bioassays (Figure 1C,D).

### 2.2. Prenylated Stilbenoid A-1 Shows the Highest Effect on Inhibition of Cell Proliferation

**Figure 2:**
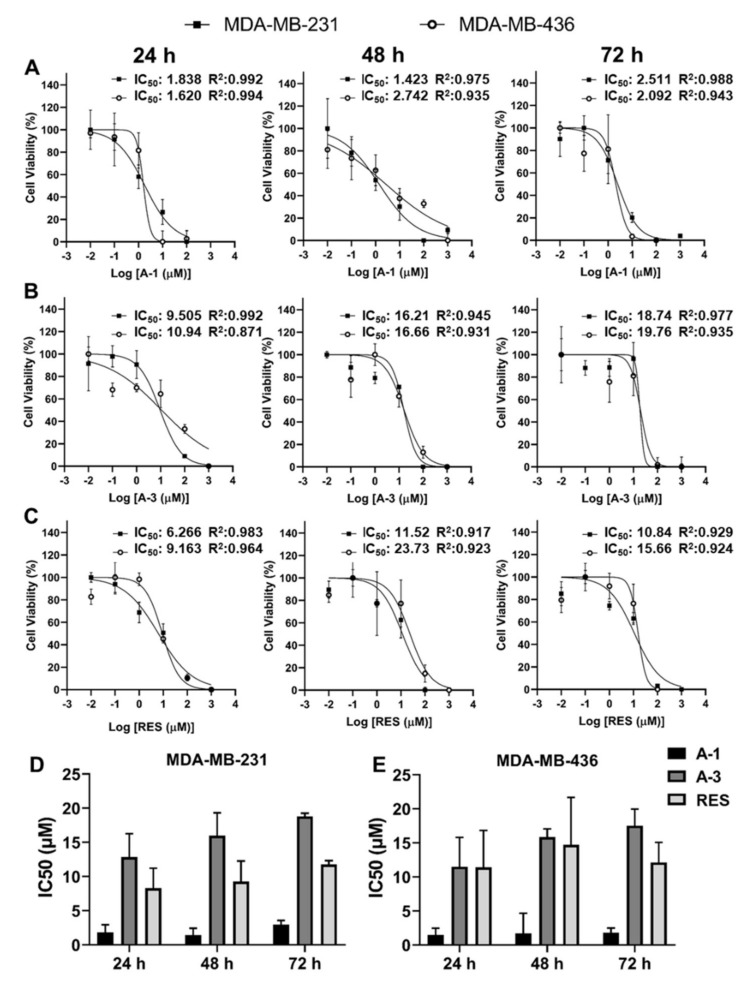
Effect of arachidin-1 (A-1), arachidin-3 (A-3), and resveratrol (RES) on cell proliferation. MDA-MB-231 and MDA-MB-436 cells were treated with various 10-fold concentrations from 0.01 µM to 1000 µM of (**A**) A-1, (**B**) A-3, and (**C**) RES for 24, 48, and 72 h. Cells with 0.1% DMSO were used as controls, wherein *n* = 3. (**D**,**E**) Summaries of the average IC_50_ values from both cell lines are displayed. Each bar represents the mean of three individual experiments ± SD.

**Figure 3:**
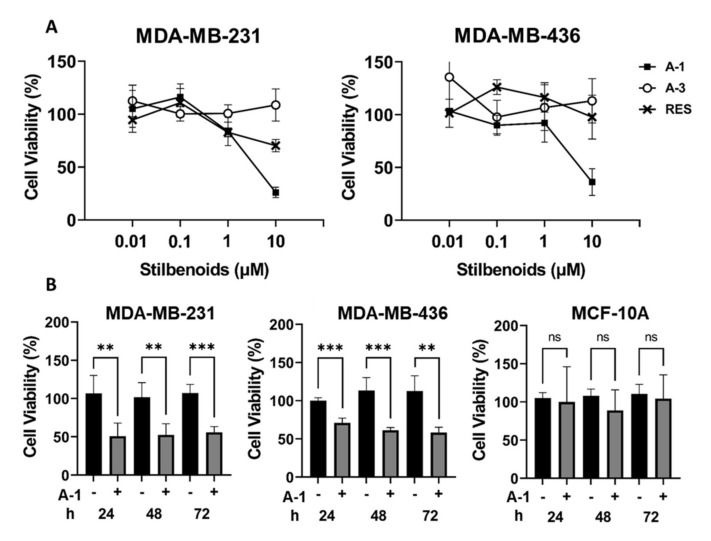
Effect of arachidin-1 (A-1), arachidin-3 (A-3), and resveratrol (RES) on growth inhibition. (**A**) MDA-MB-231 and MDA-MB-436 cells were treated with 10, 1, 0.1, and 0.01 µM of RES, A-1, and A-3 for 72 h. (**B**) Comparison of the cytotoxicity of A-1 in different cell lines. Cells were treated with 1 µM of A-1 for 24, 48, and 72 h. Cells with 0.1% DMSO were used as controls, wherein *n* = 3. Each bar represents the mean of three replicates ± SD. ** *p* < 0.001, *** *p* < 0.0001, and ns = not significant relative to control.

To evaluate the anticancer effect of A-1, A-3, and RES in two TNBC cell lines, namely MDA-MB-231 and MDA-MB-436, the IC_50_ values of each compound were measured. The cytotoxic effects of A-1, A-3, and RES varied between the two cell lines. The IC_50_ values in the MDA-MB-231 cell line of A-1, A-3, and RES were estimated to be 2.68, 18.71, and 32.07 µM after 24 h of treatment; 7.82, 16.21, and 6.54 µM after 48 h of treatment; and 2.51, 18.74, and 10.84 µM after 72 h of treatment, respectively. For cell line MDA-MB-436, the IC_50_ values of A-1, A-3, and RES were 11.95, 10.95, and 37.50 µM at 24 h; 6.20, 16.66, and 23.73 µM at 48 h; and 2.09, 19.78, and 15.66 µM at 72 h, respectively (Figure 2A–C). The results showed a reduction of cell growth by A-1, A-3, and RES in MDA-MB-231 and MDA-MB-436 cell lines; however, A-1 was found to be the most potent in both TNBC cell lines when compared to A-3 and RES (Figure 2D,E). A subsequent experiment was done with lower concentrations of A-1, A-3 and RES ranging from 0.1 µM to 10 µM to verify the more potent cytotoxic effect of A-1 among the three stilbenoids. The results indicated that A-1 had higher inhibitory growth effects at lower micromolar concentrations when compared to RES in both TNBC MDA-MB-231 and MDA-MB-436 cell lines, while A-3 showed no growth inhibition below 10 µM in TNBC cell lines after 72 h of treatment (Figure 3A). The antiproliferative effect of A-1 (1 µM; lower than A-1 IC_50_) was measured at three time points using the continually monitored Realtime Glo assay. A-1 (1 µM) showed cell proliferation reductions in MDA-MB-231 and MDA-MB-436 cells as reflected by a comparison of the difference in percentage of the cell viability of A-1-treated groups and control groups (Figure 3B). In contrast, epithelial cells (MCF-10A) did not show any statistically significant cytotoxicity when compared to control groups in all time points. These results suggested that A-1 at low concentrations specifically targeted TNBC cell lines.

### 2.3. A-1 Blocks TNBC Cell Division Cycle in G2/M Phase

**Figure 4:**
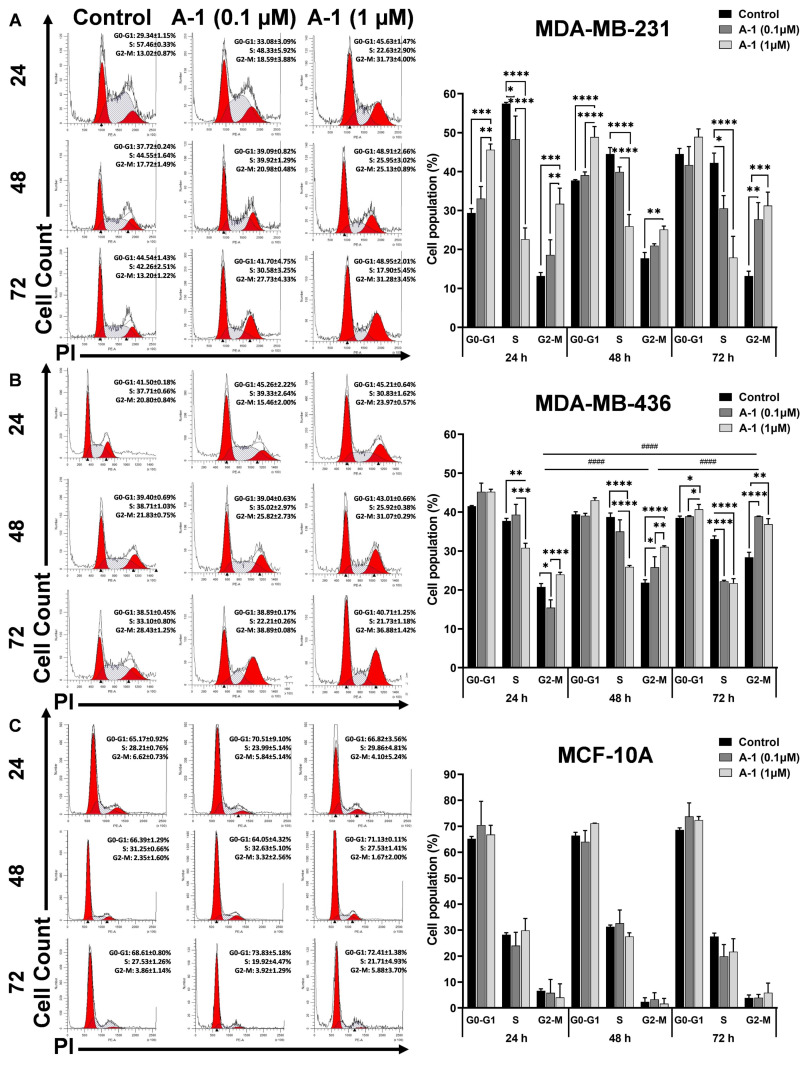
Effect of arachidin-1 (A-1) on cell cycle in MDA-MB-231 and MDA-MB-436 cells. Cells were treated with A-1 (0.1 and 1 µM) for 24, 48, and 72 h. Each histogram represents one of three experiments performed independently. (**A**) MDA-MB-231, (**B**) MDA-MB-436, and (**C**) MCF-10A-treated cells. Control groups were cells with 0.01% DMSO only. Data shown represent the mean ± SD from three replicates. * *p* < 0.05, ** *p* < 0.001, *** *p* < 0.0001 **** *p* < 0.00001 versus control, and ns = not significant from control.

The growth inhibitory effect of A-1 on TNBC cell lines was determined by flow cytometry. The results showed a statistically significant dose-dependent increase in G2-M and decrease in S phases when compared to non-treated cells in both cell lines. As shown in Figure 4A, A-1 caused cell arrest in the G2-M phase in MDA-MB-231. A significant dose-dependent increase in the percentage of cells was observed in G0-G1 cell cycle phases at 24 and 48 h, but at 72 h of treatment, it was not significant. A-1 showed about 18.59 ± 3.88% of cells (0.1 µM; ns) and 31.73 ± 4.00% (1 µM; *p* < 0.0001) in the G2-M phase. After 48 h of treatment, there were about 20.98 ± 0.48% (0.1 µM; ns) and 25.13 ± 0.89% (1 µM; *p* < 0.001) in the G2-M phase. After 72 h, the percentage of cells in the G2-M phase was 27.73 ± 4.33% (0.1 µM; *p* < 0.001) and 31.28 ± 3.45% (1 µM; *p* < 0.0001). In cell line MDA-MB-436, flow cytometry analysis also showed that treatment with A-1 increased the percentage of cells in the G2-M phase when compared to the controls. The percentage of cells in the G2-M phase increased to 23.97 ± 0.57% (1 µM; ns) after 24 h of treatment, 25.82 ± 2.73% (0.1 µM; *p* < 0.05) and 31.07 ± 0.29% (1 µM; *p* < 0.00001) after 48 h of treatment, and 38.89 ± 0.08% (0.1 µM; *p* < 0.00001) and 36.88 ± 1.42% (1 µM; *p* < 0.001) after 72 h of treatment (Figure 4B). The MDA-MB-436 cell line showed a dose and time-dependent effect of A-1 in blocking cells at G2-M. As shown in Figure 4C, there were no significant changes in the percentage of cells at different phases in A-1-treated control MCF-10A cells at the different doses and times tested. Overall, these results showed that A-1 treatment arrests cells mostly in G2-M phases while reducing the cell population in the S phase at low micromolar concentrations in the TNBC cells and also suggests that A-1 is not toxic to the non-cancerous epithelial cells at these low micromolar concentrations.

### 2.4. A-1 Induced Apoptosis in TNBC Cell Lines in a Dose-Dependent Manner

**Figure 5:**
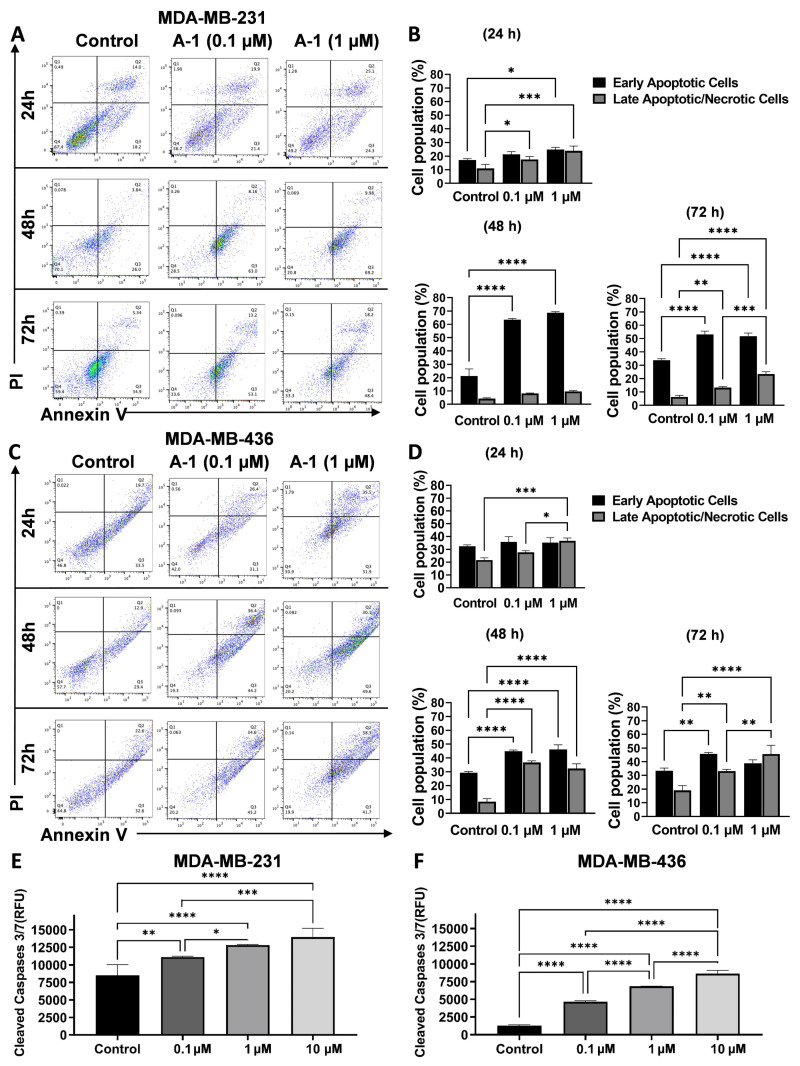
Induction of apoptosis by stilbenoids in TNBC cell lines determined by (**A**–**D**) flow cytometry and (**E**,**F**) caspase 3/7 activity. For flow cytometry assays for (**A**,**B**) MDA-MB-231 and (**C**,**D**) MDA-MB-436 cells were treated with 0.1 and 1 µM of arachidin-1 (A-1) for 24, 48, and 72 h. Early apoptosis was measured using Annexin-V/FITC/PI kits (BD Biosciences) for flow cytometry. Live cells, early apoptotic cells, and late apoptotic/necrotic cells are shown in the lower left (FITC-/PI-), lower right (FITC+/PI), and upper right (FITC+/PI+) quadrants, respectively. For caspase 3/7 activity, (**E**) MDA-MB-231 and (**F**) MDA-MB-436 cells were treated with 0.1, 1, and 10 µM of A-1 for 48 h. Caspase 3/7 activity was measured using the Apo-ONE^®^ Homogenous Caspase-3/7 Assay. Fluorescence was measured using excitation at 485 ± 20 nm and emission at 530 ± 25 nm. Cells with 0.01% DMSO were used as controls. Data represent the mean ± SD from three replicates. * *p* < 0.05, ** *p* < 0.001, *** *p* < 0.0001 **** *p* < 0.00001 versus control, and ns = not significant from control.

To confirm that the growth inhibition effect of A-1 was associated with apoptosis, apoptotic cell populations and caspase-3/7 activity were measured. As shown in Figure 5A,B, the rate of apoptosis increased in a dose-dependent manner in cell line MDA-MB-231. The percentages of cells in early apoptosis that were treated with 0.1 and 1 µM A-1 after 24 h were 21.29 ± 1.94% and 24.76 ± 1.65%, respectively, whereas the control was 17.13 ± 0.94%. After 48 h, they were 63.47 ± 0.90% and 68.67 ± 0.76%, and the control was 21.24 ± 5.34%. After 72 h, they were 53.1 ± 2.50% and 51.77 ± 2.37%, and the control was 33.69 ± 1.16% (Figure 5B). The increase of early and late apoptosis was significantly observed in MDA-MB-436 when compared with the control but the percentage of cells in early apoptosis was not statistically significant between the A-1 concentrations of 0.1 and 1 µM. The percentages of early apoptotic cells treated with 0.1 and 1 µM A-1 after 24 h were 35.76 ± 4.10% and 35.27 ± 3.78%, respectively, and the control was 32.53 ± 1.00%. After 48 h, they were 44.90 ± 0.89% and 46.11 ± 3.40%, and the control was 29.35 ± 0.86%; after 72 h, they were 45.73 ± 0.96% and 38.77 ± 2.59%, and the control was 33.34 ± 2.00% (Figure 5C,D). Therefore, the results of this study suggest that A-1 has apoptotic effects on MDA-MB-231 in a dose-dependent manner and shows significant late apoptotic effects on MDA-MB-436.

Furthermore, the apoptosis-mediated cell death mechanism was investigated by measuring caspase-3/7 activity in TNBC cell lines after 48 h treatments. The results showed a significant increase of dose-dependent caspase-3/7 activities when compared to the control (Figure 5E,F).

### 2.5. Impact of A-1 in Caspase-8 and Caspase-9, and PARP and Survivin Expression in TNBC Cell Lines

**Figure 6:**
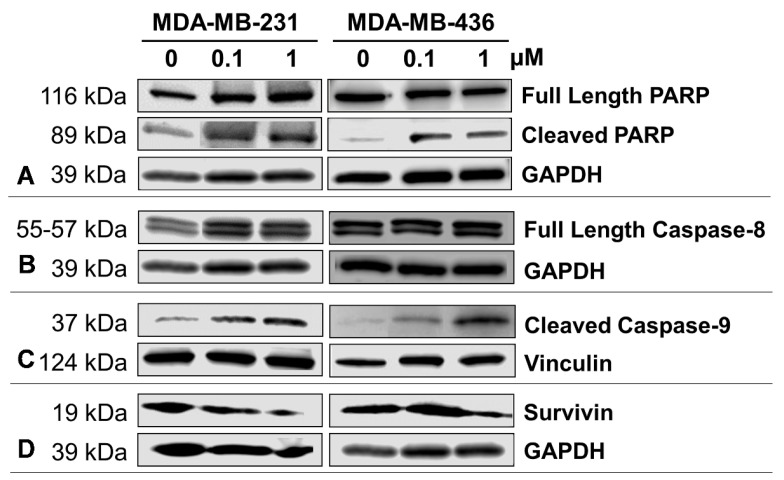

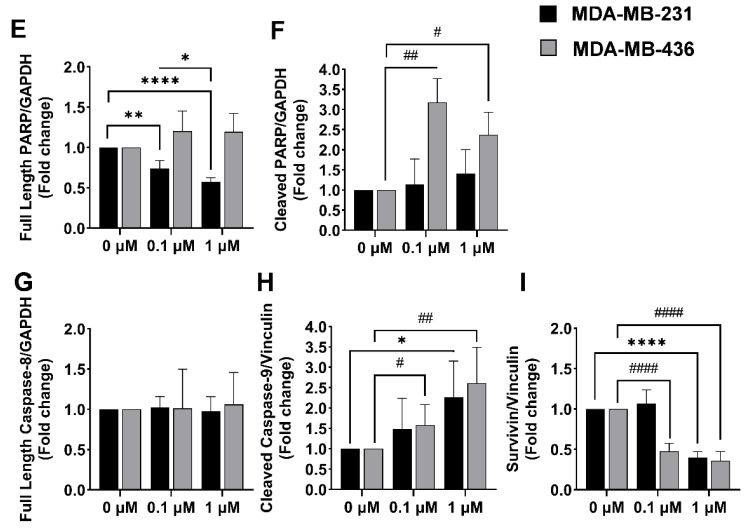
Western blot analysis and densitometry measurements of treated TNBC cell lines with arachidin-1 (A-1, 0.1, and 1 µM). Western blot images of (**A**) PARP and cleaved PARP, GAPDH loading control. (**B**) Full-length caspase-8, GAPDH loading control; (**C**) cleaved caspase-9, vinculin loading control; and (**D**) survivin, GAPDH loading control. Lysates (60 µg of total protein) of MDA-MB-231 and MDA-MB-436 cells treated with A-1 were analyzed by western blotting. The relative densitometry of (**E**) full-length PARP, (**F**) cleaved PARP, (**G**) caspase-8, (**H**) cleaved caspase-9, and (**I**) survivin protein levels were analyzed by comparing to the loading control. Cells with 0.01% DMSO were used as controls. Data represents mean ± SD from three or more independent experiments. * *p* < 0.05, ** *p* < 0.001, **** *p* < 0.0001 versus control in MDA-MB-231, ^#^ *p* < 0.05, ^##^ *p* < 0.001, ^####^ *p* < 0.00001 versus control in MDA-MB-436, and ns = not significant from control.

To verify the effect of A-1 in either extrinsic or intrinsic apoptotic pathways, the levels of caspase-8, 9, PARP, and survivin proteins were analyzed in treated TNBC cell lines by western blot. The analysis showed that A-1 treatment led to significantly decreased levels of the full-length PARP protein in MDA-MB-231 (Figure 6A,E), whereas the level of cleaved PARP was significantly increased in MDA-MB-436 but only slightly increased and not statistically significant in MDA-MB-231 (Figure 6B,C). Full-length caspase-8 levels showed no significant change in either cell lines (Figure 6B,G). The levels of cleaved caspase-9 were also significantly increased in MDA-MB-436; however, in MDA-MB-231 cells, cleaved caspase-9 levels were only significantly increased in A-1 1 µM when compared to the control group (Figure 6C,H). A dose-dependent increased in cleaved caspase-9 was observed in both cell lines. Furthermore, survivin expression was downregulated in both TNBC cell lines (Figure 6D,I). These results suggest that A-1 induced apoptosis in MDA-MB-231 and MDA-MB-436 through the intrinsic pathway.

## 3. Discussion

TNBC cancer consists of 10 to 15% of all breast cancer types and is an aggressive type of cancer due to its quick cell proliferation and metastasis [38]. Since hormonal and targeted therapies are ineffective in treating TNBC, chemotherapy is the most common treatment [38]. Theoretically, chemotherapeutic drugs target the metastatic cells, but no treatment can specifically target TNBC cells. Additionally, chemotherapeutic drugs also have long-term side effects and high toxicity in non-cancerous cells [39]. Therefore, in this study, we investigated plant-made natural products as potentially alternative therapeutics.

According to the World Health organization, around 80% of people around the world use natural medicine, and based on the high percentage of mortality caused by cancer, studying natural products which can target TNBC cells is fundamental [40,41]. An interesting point is that 60% of all natural products have been used to treat cancer [42]. Several studies have proposed that RES, a natural product found in taxonomically unrelated plant species such as grape and peanut, can be a potential anti-cancer drug or cancer preventive supplement. Indeed, RES has been shown to induce apoptosis in TNBC cell lines like MDA-MB-231 [43,44]. However, RES has low bioavailability due to extensive metabolism [45], thus high concentrations have been required to show effective results in clinical trials. Prenylated stilbenoids such as A-1 and A-3 share the same stilbene backbone as RES and may experience RES-like properties in vitro with potentially high bioavailability in vivo [32]. Enzymatic assays have shown that the presence of the prenyl group in their structure prevents action by UDP-glucuronosyltransferases involved in phase II drug metabolism. Accordingly, prenylated stilbenoids have shown more metabolic stability when compared to their non-prenylated analogs [32]. Herein, RES and two prenylated analogs, namely A-1 and A-3, were studied and A-1 was identified as a potent cytotoxic stilbenoid in TNBC cells. Importantly, the A-1 showed low cytotoxicity to the non-cancerous epithelial cells.

Studies with plant-made prenylated stilbenoids have been limited because these compounds accumulate at very low levels in plant tissues and, in most cases, only under certain stress conditions. Furthermore, these compounds are not commercially available. In this study, a peanut hairy root culture, which can be manipulated to produce and accumulate prenylated stilbenoids in the medium, was used as a bioproduction for the prenylated stilbenoids A-1 and A-3. Upon elicitation, the combined levels of A-1 and A-3 in the culture medium can reach approximately 600 mg/L [36]. After a simple organic extraction from the culture medium, extracts rich in prenylated stilbenoids were obtained and subjected to semi-preparative HPLC to obtain highly purified compounds for bioassays. Previously, A-1 and A-3 were produced in fungal-challenged peanut seeds and extensive purification steps were needed to purify these compounds as well as remove potential fungal toxins [46]. The hairy root-based bioproduction platform in the current study provides a sustainable axenic system for A-1 and A-3 production.

This study reports the inhibitory effects of A-1 and A-3 on the cell proliferation of MDA-MB-231 and MDA-MB436. A-1, A-3, and RES were primarily examined for cytotoxicity effects on these TNBC cell lines. Although other studies showed that a high concentration of RES was required to inhibit 50% of cell proliferation in breast cancer cells (MCF-7 cells IC_50_: 131 μM after 24 h treatment and IC_50_: 83.9 μM after 48 h treatment; MDA-MB-231 IC_50_: 144 μM after 24 h treatment by performing MTT assay) [47,48], we observed that low concentrations of RES and RES analogs (A-1 and A-3) were needed to kill 50% of MDA-MB-231 and MDA-MB-436 cells by using continually monitored Realtime Glo assays (Promega, Madison, WI, USA). The results indicated that A-1 had the highest cytotoxicity effect in all treatment time points, with an IC_50_ of about 2 μM for both cell lines, and the low concentrations of A-1 had no significant cytotoxicity effects on the normal human breast epithelial cell line MCF-10A. This result agrees with previous findings, which reported that A-1 inhibited cell proliferation at lower concentrations in the human leukemia HL-60 cell line when compared to A-3 and RES, and had no cytotoxic effects on peripheral blood mononuclear cells (PBMCs) [34]. The IC_50_ values in MDA-MB-231 and MDA-MB-436 cell lines after 72 h treatment were 2.51 and 2.09 μM for A-1; 18.74 and 19.76 μM for A-3; and 10.84 and 15.66 μM for RES, which are similar to a previous study with the human leukemia HL-60 cell line where the IC_50_ values of A-1, A-3 and RES after 72 h treatment were 4.2, 18.9, and 17.6 μM, respectively (Figure 2A–C) [34]. Other studies presented similar results for A-1, which showed the greatest cytotoxicity on three other cancer cell lines: skin, breast, and ovarian [49]. Our results revealed that the IC_50_ of A-1, A-3, and RES was not time-dependent, which is supported by studies with several cancer cell lines. For instance, one study which evaluated the effect of different concentrations of an RES analog (SS28; 1, 5, 10 and 20 μM) on various leukemic cell lines (CEM, Reh, Molt4, and Nalm6), human lung carcinoma cells (A549), the cervical cancer cell line (HeLa), the diffuse large B cell lymphoma cell line (SUDHL8), and the human embryonic kidney epithelial cell line (HEK293T) for 48 and 72 h did not see a time-dependent change in cell viability after treatment with SS28. [50].

A-1 has been shown to induce apoptosis by changing cellular morphology and chromatin condensation. To detect changes in the cellular morphology resulting from apoptosis, flow cytometry with Annexin V/PI staining was used to quantify early and late apoptotic cells. The Apo-ONE^®^ Homogeneous Caspase-3/7 Assay (Promega, Madison, WI, USA) was used to detect caspase-3 and caspase-7 activation, which are the hallmarks of apoptosis that initiate DNA fragmentation. The data showed that A-1 induced apoptosis in MDA-MB-231 and MDA-MB-436 in dose-dependent manners, leading to an increase in the early and late apoptotic cells. A-1 led to a statistically significant increase in the cell population percentage in early apoptosis in a dose-dependent manner at all treatment time points and only slight and not significant increases were observed in the late apoptotic cell population in MDA-MB-231 cells. The results showed a statistically significant increase in the cell population percentage of early and late apoptosis for all hours in MDA-MB-436 cells. This study showed a meaningful increase in caspase-3/7 activities by A-1 treatment at 24 h in a dose-dependent manner. The results are in agreement with a previous study where A-1 induced apoptosis in a dose-dependent manner (0 to 4 μM) in human leukemia HL-60 cells after 24 h by activation of caspase-3 and 9 [34]. Other similar studies have shown that RES induced early apoptosis in a dose-dependent manner after 24 h in MCF-7 breast cancer cells and HepG2 liver cancer cells [29,51]. However, RES showed an increase in the population of cells in late apoptosis in U937 and MOLT-4 leukemia cells in a dose-dependent manner [51]. RES also showed an increase in apoptosis in a dose-dependent manner in MIA PaCa-2 pancreatic cancer cells [52]. The current study confirmed that A-1 at low concentrations could inhibit TNBC cell proliferation and induce apoptosis. Chromatin fragmentation or DNA damage is a molecular event that correlates with cell cycle arrest and induction of apoptosis. The cell cycle arrest occurs when cells are under stress or have damaged DNA. Therefore, the cell undergoes cell cycle arrest to have more time to repair the DNA. When DNA damage is intense, the cell goes through apoptosis [53].

In this study, A-1 treatment resulted in an accumulation of cells in G2-M phase, indicating that A-1 inhibits the cellular proliferation due G2-M phase cell cycle arrest in a dose-dependent manner in MDA-MB-231 and MDA-MB-436 cells. Several studies agree with these results. For instance, RES induced in a dose-dependent manner cell cycle arrest in the G2-M phase after treating cancer cells for 24 h [54]. These results indicated that A-1 inhibits proliferation and induces apoptosis, which may have derived from either intrinsic or extrinsic apoptosis pathways.

Several studies have shown that resveratrol, piceatannol, and resveratrol analogs induce apoptosis in breast cancer cells [29,48,55,56,57]. This research demonstrated the apoptotic effect of A-1 in MDA-MB-231 and MDA-MB-436 cells (Figure 5). In order to examine the apoptosis mechanism induced by A-1 in TNBC cell lines, we studied the protein levels of full-length PARP, cleaved PARP, which is activated by caspase-3, and survivin, an apoptosis inhibitor. We found that A-1 induced apoptosis in both TNBC cell lines by activating PARP cleavage and inhibiting survivin expression. RES in several studies showed the inhibition of survivin and cleavage of PARP. Other studies indicated that RES induced apoptosis in various cancer cell lines and TNBC cell lines through extrinsic and intrinsic pathways by activating caspase-8 and 9; however, the major pathway of RES is intrinsic through activation of caspase-9-dependent mitochondria pathways [26,27,28,29,30]. A-1 induced apoptosis through caspase-dependent and caspase-independent pathways through mitochondria depolarization. We also observed a similar finding that A-1 activates the mitochondrial caspase-9 pathway [34]. Caspases are an essential feature for targeting the potential therapeutics for cancer due their cell regulation and apoptosis initiation functions. Both the MDA-MB-231 and MDA-MB-436 cell lines exhibited capase-9 activation. The higher hydrophobicity of A-1 due to the presence of a prenyl group, in comparison with RES, might be the reason it interacts more with the mitochondrial membrane and activates caspase-9 [34].

## 4. Materials and Methods

### 4.1. Growth of Hairy Roots and Elicitation of Stilbenoids

Peanut hairy roots (cultivar Hull line 3A) were maintained in 250 mL flasks with 50 mL of modified Murashige and Skoog medium (MSV) [35] on an orbital shaker at 90 rpm, at 28 °C, and in complete darkness. After 9 days of culture, the medium was replaced with elicitation medium and the hairy roots continued to be cultured for 192 h. The elicitation medium consisted of 100 mL of MSV medium with 125 µM of methyl jasmonate (Sigma-Aldrich; St. Louis, MO, USA), 3 mM of H_2_O_2_ (Thermo Fisher Scientific; Waltham, MA, USA), 18 g/L of methyl-β-cyclodextrin (CD; CAVASOL^®^ W7 M, Wacker, Munich, Germany), and additional 1 mM f MgCl_2_ (Sigma-Aldrich; St. Louis, MO, USA) as described before [36].

### 4.2. Extraction and Purification of Stilbenoids by Semi-Preparative HPLC

The prenylated stilbenoids A-1 and A-3 were isolated from extracts of elicitor-treated hairy root cultures of peanut as described before [36]. After 192 h of elicitation, stilbenoids were extracted from the culture medium by partitioning with ethyl acetate (Thermo Fisher Scientific; Waltham, MA, USA) twice. Ethyl acetate extracts from several cultures were combined and dried in a rotary evaporator (Buchi; Rotavapor R-200; Flawil, Switzerland). The dried extract was resuspended in methanol (Thermo Fisher Scientific; Waltham, MA, USA) at a concentration of 100 to 150 mg/mL and then 100 μL was injected to the semi-preparative HPLC column for further purification. The separation was performed on a semi-preparative column (SunFire C18, 5 μm, 10 × 250 mm, Waters; Milford, MA, USA) at room temperature in an UltiMate 3000 LC system (Thermo Fisher Scientific; Waltham, MA, USA). The mobile phase consisted of water (A) and methanol (B) at a 4 mL/min flow rate. The column was initially equilibrated with 60:40 (A:B). Then, solvent B was linearly increased to 50% for 2 min (0−2 min) and then further increased to 70% for 48 min (2–50 min) as well as held at 100% for 5 min (50–55 min). The mobile phase was returned to the initial condition of 60:40 (A: B) for an additional 5 min. The fractions of the targeted stilbenoids A-1 and A-3 were collected based on the real-time UV chromatogram, dried in a SpeedVac, and weighed with an analytical balance. The collected stilbenoid fractions were analyzed by analytical HPLC as described before [37]. Compounds purified to greater than 96.5% as determined by analytical HPLC were used. The identity of A-1 and A-3 was previously confirmed in the peanut hairy root cultures by mass spectrometry and by comparison to authentic standards purified from elicited peanut seeds [35]. RES standard was purchased from Sigma-Aldrich. RES, A-1, and A-3 were dissolved in dimethyl sulfoxide (DMSO) (ATCC; Manassas, VA, USA) for cell culture assays.

### 4.3. Culture of Human Cell Lines

The epithelial TNBC cell lines MDA-MB-231 (ATCC HTB-26™; Manassas, VA, USA) and MDA-MB-436 (ATCC HTB-130™; Manassas, VA, USA), and the normal breast epithelial cell line MCF-10A (ATCC CRL-10317™; Manassas, VA, USA), were purchased from the American Type and Culture Collection (ATCC; Manassas, VA, USA). The MDA-MB-231 cells were cultured in Dulbecco’s Modified Eagle’s Medium (DMEM) (ATCC; Manassas, VA, USA) supplemented with 10% fetal bovine serum (HyClone; Logan, UT, USA) and 1% antibiotic (100 IU/mL of penicillin and 100 µg/mL of streptomycin; HyClone; Logan, UT, USA), and incubated at 37 °C, with a humidified atmosphere of 5% CO_2_. The MDA-MB-436 cells were cultivated in Leibovitz’s L-15 medium (ATCC; Manassas, VA, USA) supplemented with 10% fetal bovine serum and 1% penicillin–streptomycin solution (100 IU/mL of penicillin and 100 µg/mL of streptomycin; ATCC; Manassas, VA, USA), 10 µg/mL of insulin (Gibco; Life Technologies; Grand Island, NY, USA), and 16 µg/mL of glutathione (Sigma-Aldrich; St. Louis, MO, USA), and maintained at 37 °C, with a humidified atmosphere of 0% CO_2_. The MCF-10A cells were cultured in DMEM/F12 medium supplemented with a MEGM Kit (Lonza Pharma and Biotech; Basel, Switzerland) and incubated at 37 °C, with a humidified atmosphere of 5% CO_2_.

### 4.4. Cytotoxicity Assay

The cytotoxicity of RES, A-1, and A-3 was measured using the RealTime-Glo^TM^ MT Cell Viability Assay (Promega, Madison, WI, USA). The cells (10,000 cells/well) were seeded into 96-well plates. After 24 h, they were treated with various 10-fold dilutions of RES, A-1, and A-3 with concentrations ranging from 0.1 µM to 1000 µM. Cells with 0.1% DMSO (ATCC; Manassas, VA, USA) were included as a control. Luminescence was measured every 12 h for 72 total hours in a Cytation^TM^ 5 (BioTek, Winooski, VT, USA) plate reader. All experiments were performed in triplicates. The IC_50_ (concentration of compound exhibiting 50% of cell growth inhibition) was attained from standard curves of the cell viability percentage vs. log concentration of compounds using GraphPad Prism 9(San Diego, CA, USA). Based on the IC_50_ of each compound and lower toxicity to MCF-10A, the most effective compound was subjected to further examination.

### 4.5. Cell Cycle Analysis with PI

Cells were seeded onto 6-well plates and treated with A-1 (0.1 µM and 1 µM) for 24, 48, and 72 h. Cells incubated with 0.01% DMSO (ATCC; Manassas, VA, USA) were used as a control. Then, MDA-MB-231 and MCF-10A cells were collected by trypsinization; MDA-MB-436 cells were collected by scraping. Treated cells were fixed with ice-cold 70% ethanol while vortexing and incubated for 1 h in 4 °C. Then, cells were washed with cold 1X DPBS, treated with 1 mg/mL of ribonuclease A, and incubated for 30 min at 37 °C. The cells were then stained with 10 µg/mL of propidium iodide (PI), incubated for 30 min at 37 °C, and analyzed by flow cytometry using BD FACS Aria Sorter (BD Biosciences, Franklin Lakes, NJ, USA). Data were analyzed with Modfit LT 5.0 software (Verity Software House; Topsham, ME, USA). All experiments were performed in triplicates.

### 4.6. Analysis of Apoptosis Using Annexin V-FITC Assay

Cells (10^6^ cells/well) were seeded onto 6-well plates. After 24 h, the cells were incubated with A-1 (0.1 and 1 µM) for 24, 48, and 72 h. The control groups were incubated with 0.01% DMSO (ATCC; Manassas, VA, USA) for every hour. Then, cells were collected and washed twice with cold 1X PBS and stained with both annexin V-FITC and PI for 5 min at room temperature. The cells were analyzed by flow cytometry using BD FACS Aria (BD Biosciences, Franklin Lakes, NJ, USA). The positive controls (annexin negative/PI negative; annexin negative/PI positive; and annexin positive/PI negative) were used to set up compensation and set quadrants. The early apoptotic cells were measured in the quadrant of annexin V positive/PI negative and late apoptotic/necrotic cells were measured in the quadrant of annexin positive/PI positive. Data were analyzed with FlowJo™ v10.8 Software (BD Life Sciences; Ashland, OR, USA). All experiments were performed in triplicates.

### 4.7. Measuring of Caspase-3/7 Activity

The Apo-ONE^®^ Homogenous Caspase-3/7 Assay (Promega, Madison, WI, USA) was used for measuring caspase-3/7 activity. Cells (10,000 cells/well) were seeded in 96-well plates. After 24 h, the cells were treated with A-1, A-3, and RES, and incubated for an additional 48 h at 37 °C, with a humidified atmosphere of 5% CO_2_ (however, MDA-MB-436 cells were incubated in 0% CO_2_). Control groups were cells treated with 0.01% DMSO (ATCC; Manassas, VA, USA). According to the manufacturer, after 48 h of treatment, the caspase reagent was added to the cells and the plates were incubated at room temperature in the dark for 1 h. Then, fluorescence was measured every hour for a total of 4 h using the Cytation^TM^ 5 (BioTek; Winooski, VT, USA) plate reader at an excitation wavelength of 485 ± 20 nm and emission of 530 ± 25 nm.

### 4.8. Immunoblotting of Caspase-8 and Caspase-9

MDA-MB-231 and MDA-MB-436 cells (10^7^ and 5 × 10^6^ cells/3 mL medium, respectively) were seeded in a 6-well cell culture plate. After 24 h, the cells were treated with A-1 (0.1 µM and 1 µM) for 24 h. Cells treated with 0.01% DMSO (ATCC; Manassas, VA, USA) were included as a control. Protein extracts were prepared using RIPA Lysis and Extraction Buffer plus Halt™ Protease Inhibitor Cocktail (Thermo Fisher Scientific; Waltham, MA, USA). The protein concentration was measured using the Bradford assay (Thermo Fisher Scientific; Waltham, MA, USA). Protein extracts (60 µg) with 5 µL of 2X Novex™ Tris-Glycine SDS Sample Buffer (Thermo Fisher Scientific; Waltham, MA, USA) and 1% dithiothreitol (DTT; Thermo Fisher Scientific; Waltham, MA, USA) were denatured and separated by electrophoresis using SDS-PAGE in 10% Tris-Glycine pre-cast gel (Novex™ WedgeWell™, Thermo Fisher Scientific; Waltham, MA, USA). The gel was run in 1X Novex™ Tris-Glycine SDS running buffer (Thermo Fisher Scientific; Waltham, MA, USA) at a constant 125 voltage for 90 min. The proteins were transferred into Trans-Blot Turbo Mini 0.2 µm PVDF membranes (Bio-Rad Laboratories; Hercules, CA, USA) using the Trans Blot Turbo System (Bio-Rad Laboratories; Hercules, CA, USA) at 1.3 amp and up to 25 volts for 7 min. After the membranes dried completely, they were incubated in 1X Tris Buffered Saline (TBS; Bio-Rad; Hercules, CA, USA) for 5 min at room temperature. Then, the membranes were blocked with 5% non-fat dry milk in 1X TBS containing 1% Tween 20 (TBST; Bio-Rad; Hercules, CA, USA) for 1 h at room temperature. The membranes were incubated with specific primary antibodies at 4 °C overnight. The primary antibodies and dilutions included rabbit anti-caspase-8 (D35G2) mAb (1:500; Cell Signaling Technology; Danvers, MA, USA), rabbit anti-PARP mAb (1:500; Cell Signaling Technology; Danvers, MA, USA), rabbit anti-human survivin polyclonal antibody (0.5 µg/mL; R&D Systems; Minneapolis, MN, USA), mouse anti-human caspase-9 mAb (1 µg/mL; R&D Systems; Minneapolis, MN, USA), mouse anti-vinculin antibody (2 µg/mL; R&D Systems; Minneapolis, MN, USA), and mouse anti-human GAPDH antibody (0.05 µg/mL; R&D Systems; Minneapolis, MN, USA). Afterwards, the membranes were incubated with HRP-conjugated goat anti-rabbit IgG secondary antibody (1:2000; Cell Signaling Technology; Danvers, MA, USA) and HRP-conjugated AffiniPure goat anti-mouse IgG secondary antibody (1:50,000; Jackson Immunoresearch Laboratories; West Grove, PA, USA) for 1 h at room temperature. The membranes were washed with 1X TBST and then incubated with SuperSignal™ West Pico PLUS Chemiluminescent Substrate (Thermo Fisher Scientific; Waltham, MA, USA). The membranes were scanned and analyzed using the Li-COR Odyssey^®^ Fc Imaging System (LI-COR Biosciences; Lincoln, NE, USA).

### 4.9. Statistical Analysis

All values were represented as the mean ± standard deviation (S.D.). Students’ t-test analyses were used when the mean difference between 2 groups was measured (Figure 3) and one-way analysis of variance (ANOVA) was used when the significance of more than two groups was compared (Figure 4, Figure 5 and Figure 6). GraphPad Prism 9 (San Diego, CA, USA) was used for all statistical analyses. Calculations where the *p*-value was < 0.05 were considered as statistically significant.

## 5. Conclusions

In conclusion, we showed that A-1 promoted apoptosis through the intrinsic pathway and reduced cell viability in TNBC, while having no toxicity impact in epithelial cells. We also observed that A-1 at low micromolar concentrations reduced cell viability and changed the cell cycle, which led to cell apoptosis. Accordingly, the ability of A-1 to suppress cell proliferation by arresting cell cycle in G0-G1 and G2-M phases, activating caspase-3 and 7, increasing cleaved PARP protein levels, and activating the intrinsic pathway in both TNBC cells supports that A-1 can be efficient at triggering programmed cell death. These findings collectively showed that A-1 merits further research as a useful compound for treating TNBC.

## Data Availability

The data of this study are available upon request.
